# School-based intervention on healthy behaviour among Ecuadorian adolescents: effect of a cluster-randomized controlled trial on screen-time

**DOI:** 10.1186/s12889-015-2274-4

**Published:** 2015-09-22

**Authors:** Susana Andrade, Maïté Verloigne, Greet Cardon, Patrick Kolsteren, Angelica Ochoa-Avilés, Roosmarijn Verstraeten, Silvana Donoso, Carl Lachat

**Affiliations:** Food Nutrition and Health Programme, Universidad de Cuenca, Avenida 12 de Abril y, Loja 010202 Cuenca, Ecuador; Department of Food Safety and Food Quality, Ghent University, Coupure links 653, 9000 Ghent, Belgium; Department of Movement and Sport Sciences, Ghent University, Watersportlaan 2, 9000 Ghent, Belgium; Institute of Tropical Medicine, Nationalestraat 155, 2000 Antwerp, Belgium

**Keywords:** Randomised controlled trial, Adolescents, Questionnaires, Sedentary behaviours, Screen-time, Computer, Video games

## Abstract

**Background:**

Effective interventions on screen-time behaviours (television, video games and computer time) are needed to prevent non-communicable diseases in low- and middle-income countries. The present manuscript investigates the effect of a school-based health promotion intervention on screen-time behaviour among 12- to 15-year-old adolescents. We report the effect of the trial on screen-time after two stages of implementation.

**Methods:**

We performed a cluster-randomised pair matched trial in urban schools in Cuenca-Ecuador. Participants were adolescents of grade eight and nine (mean age 12.8 ± 0.8 years, n = 1370, control group *n* = 684) from 20 schools (control group *n* = 10). The intervention included an individual and environmental component tailored to the local context and resources. The first intervention stage focused on diet, physical activity and screen-time behaviour, while the second stage focused only on diet and physical activity. Screen-time behaviours, primary outcome, were assessed at baseline, after the first (18 months) and second stage (28 months). Mixed linear models were used to analyse the data.

**Results:**

After the first stage (data from *n* = 1224 adolescents; control group *n* = 608), the intervention group had a lower increase in TV-time on a week day (β = −15.7 min; *P* = 0.003) and weekend day (β = −18.9 min; *P* = 0.005), in total screen-time on a weekday (β = −25.9 min; *P* = 0.03) and in the proportion of adolescents that did not meet the screen-time recommendation (β = −4 percentage point; *P* = 0.01), compared to the control group. After the second stage (data from *n* = 1078 adolescents; control group *n* = 531), the TV-time on a weekday (β = 13.1 min; *P* = 0.02), and total screen-time on a weekday (β = 21.4 min; *P* = 0.03) increased more in adolescents from the intervention group. No adverse effects were reported.

**Discussion and Conclusion:**

A multicomponent school-based intervention was only able to mitigate the increase in adolescents’ television time and total screen-time after the first stage of the intervention or in other words, when the intervention included specific components or activities that focused on reducing screen-time. After the second stage of the intervention, which only included components and activities related to improve healthy diet and physical activity and not to decrease the screen-time, the adolescents increased their screen-time again. Our findings might imply that reducing screen-time is only possible when the intervention focuses specifically on reducing screen-time.

**Trial registration:**

Clinicaltrials.gov identifier NCT01004367.

## Background

Adolescents who spend more than 2 h/day on screen-time behaviour (television (TV) viewing, computer use and video games playing) are more likely to have unfavourable body composition, low fitness and a decreased academic achievement [1]. Despite this, the prevalence of spending more than 2 h/day on screen-time behaviours among adolescents exceeds 30 % in most of the countries around the world [2, 3]. In Latin-America this prevalence reaches levels higher than 50 % [3–5].

School-based interventions have been developed to decrease screen-time [6] and resulted in a significant but small decrease in screen-time among adolescents [7]. Interventions that aimed to improve either physical activity or screen-time have reported inconsistent [8–10] or marginal effects [9, 11] on screen-time. Studies that focused both on physical activity and screen-time behaviour have shown a modest effect on screen-time behaviours [12–16]. Most of these studies however, are conducted in high-income countries. To our knowledge, studies on screen-time from low- and middle-income countries are mainly observational, underpowered or with a short follow-up period [1, 7].

In Ecuador, a comprehensive school-based intervention programme “ACTIVITAL” was conducted to improve diet and physical activity and to decrease screen-time in adolescents of 12–15 years old. The trial was delivered in two different stages. The first stage focused on diet, physical activity as well as screen-time behaviour and the second stage only focused on diet and physical activity. The intervention effects on the dietary behaviour (*Ochoa-Avilés* submitted) and physical activity and fitness [17] are documented elsewhere. These results showed that the programme was able to improve physical fitness, minimize the decline in physical activity and to improve the fruit, vegetable and whole grain intake of the adolescents. The aim of the present manuscript was to investigate the effect of the “ACTIVITAL” intervention on adolescents’ television time, videogames time, computer time and total screen-time. In addition, given the differences in implementation focus, we investigated the effect on screen-time after each stage of the intervention.

## Methods

### Procedure

ACTIVITAL was a pair-matched cluster randomised controlled trial performed in adolescents from urban schools of Cuenca, Ecuador (2009–2012). The trial was registered on ClinicalTrials.gov database under the N° NCT01004367 and was approved by both the Ecuadorian (Central University of Ecuador, code N°: CBM/cobi-001 – 2008/462) and Belgian (Ghent University Hospital, code N°: FWA00002482) ethics committee. Twenty schools were randomly selected from a list of eligible schools. Meetings were organized with the principals of each school, adolescents and their caretakers to inform them about the project. All principals agreed to participate with their school and 85 % of adolescents and their caretakers signed an informed assent and consent. Further details about the exclusion criteria, sample size calculation, allocation, blinding and randomization procedure are described elsewhere [17].

#### Participants and intervention

A total of 1440 adolescents (control group *n* = 740), from 20 urban schools (control group *n* = 10), participated in the trial (62.4 % female, mean age 12.8 ± 0.8 years). The intervention objective and strategies were developed using the results of previously conducted quantitative (cross-sectional research) [18] and qualitative (focus groups) [19] studies jointly with the Intervention Mapping protocol [20] and Comprehensive Participatory Planning and Evaluation approach [21].

The intervention programme comprised two stages. Each stage included both individual and environmental oriented strategies implemented by the school staff or the researchers. This manuscript only includes information on the strategies related to physical activity and sedentary behaviour, as these were linked in the intervention programme. In both stages the individual strategy was delivered by means of an educational package which was implemented at classroom level by school staff and researchers. The educational package involved a textbook for teachers and workbook for adolescents. The environmental strategies were designed to increase the opportunities of the adolescents to be active and reduce the screen-time. The environmental strategies, in both intervention stages, included modifications of the school environment and one parental workshops conducted in parallel to the classes with adolescents and with similar topics.

In the first stage of intervention the individual strategy was oriented to two key messages regarding physical activity and screen-time behaviour: i) be active for at least 60 min/day, and ii) spend maximum 2 h/day on watching TV. These two key messages were also tackled on the parental workshop (environmental strategy). The parental workshop consisted of a slide show followed by a session of questions for parents. Besides, as part of the environmental strategy pep talks with famous young sportsmen and – women were organized. During the pep talks, the sportsmen and – women shared their experiences about their life style, encouraged adolescents to be active and answered questions of the adolescents about their life style (Table 1).

**Table 1:** Description by stages of the ACTIVITAL strategies^a^

INTERVENTION STRATEGIES FOR STAGE 1				
	What	When /Who/Where	Why	How
Individual-based strategies	One textbook was included in the curriculum: Book 1 The book contained five chapters, and one (chapter2) of them addressed physical activity and TV time. This chapter was developed to be delivered in a lesson of 90 min	The content of book was taught from September 2010 - February 2011 Each chapter was taught every two weeks./ School teachers and trained staff/ in the classroom	- To introduce the notion that more than 2 h on TV/day is not healthy. - To create awareness regarding the importance of an adequate physical activity throughout adolescence - To increase knowledge and enhance decision making skills	The textbooks and pedagogic materials were developed for teachers and students. The textbooks for teachers contained educational objectives, clear instructions for implementing the physical (ludic games) and educational activities during the classes without additional training and theoretical information. The textbooks of adolescents were a workbook and contained theoretical information.
Environment-based strategies	Workshop with parents ------------------------------------ One workshop was performed and focused on decreasing TV time and increasing physical activity. The workshop included the distribution of informative leaflets to each participant. Each leaflet included theoretical information, advices and benefits on the particular topic of the workshops	The workshops were performed from October 2010 till February 2011/ the ACTIVITAL staff delivered the workshop/in the school meeting room	To increase the awareness of parents regarding the importance of decreasing TV time and of regular physical activity for adolescents. - To support healthy behaviour regarding the physical activity and TV time of adolescents at home	The parents were summoned through a letter signed by each school principal. The workshop lasted 1 h. ACTIVITAL staff gave a presentation about the main messages during the first 35min of the workshop. Afterwards the parents could ask questions to clarify the messages, and finally the leaflets were distributed.
	Social event	One pep talk was organized during the intervention/the young athletes were incharge of the pep talks/in the school auditorium	To encourage physical activity through the positive influence of social models	A 1 hour interactive session with young athletes was organized. Athletes shared their personal sport experiences and gave advice on an active lifestyle and physical activity.
	Pep talks were organized in each school. The pep talks had the participation ofsuccessful and well-knownyoung male (n = 3) and female (n = 2) athletes, whowere international young champions in BMX, swimming, racquetball and weight lifting.			
	Posters for classroom and food tuck shop	The poster were suspended monthly from October 2010 to February 2011/the ACTIVITAL staff suspended the poster/on the walls of classroom and food trucksshop	- To encourage students to be active and eat healthy	Posters including key messages to be active were suspended on the classroom walls and in front of the food tuckshops. Not all poster were suspended on the walls at once: A new poster was suspended on the walls each month.
	Five different posters with pictures of young athletes and key messages to promote physical activity and healthy eating behaviours.			
INTERVENTION STRATEGIES FOR STAGE 2				
Individual-based strategies	One textbook was included in the curriculum: Book 2 The book contained 8 chapters in total and one (chapter 8) corresponded to the physical activity. This chapter was focused on how to remove barriers in order to be more physically active. This chapter was planned to be though in 90 min.	The lessons were delivered from September 2011-January 2012. Each chapter was taught every two weeks (i.e. 1 lesson every two weeks). /School teachers and trained staff delivered the lessons / in the classroom	- To give ideas on how to deal with barriers to be physically active. - To increase knowledge and enhance decision making skills	A second set of textbooks and pedagogic materials were developed for teachers and students. The textbooks of teachers contained educational objectives, clear instructions for implementation the physical and educational activities during the classes without additional training and theoretical information. The textbooks of adolescents were workbooks and contained theoretical information.
Environment-based strategies	Parental workshops _________________________________ One workshop about dealing with barriers for physical activity was organized. Informative leaflets supporting the content of the workshop were distributed to each participant of workshop	The workshops were performed from October 2011 till January 2012 /the ACTIVITAL staff was in charge of the workshop/in the school meeting room	- To give ideas on how to deal with barriers to be physically active. - To support healthy behaviour regarding to how to overcome the barriers to be physically active at home	Parents’ attendance was mandatory through a letter signed by each school principal The workshop was delivered in 1 hour. Each leaflet included theoretical information, advices and benefits on the particular topic of the workshops.
	Walking trail and posters - Using line markings, a walking trail was drawn on the school’s playground. The length of the trail was the perimeter of playground. - 3 posters suspended on the school walls adjacent to the trail, with phrases like: “Do you like to talk? Walk and Talk”	The walking trails on each school were implemented on September 2011, and were used between September 2011 – January 2012 until the marks disappear because of the rain/the physical education teachers explained the objective of walking trails to students/on the school’s playground	- To increase the availability and accessib ility to opportunities for physical activity inside the schools - To motivate the students to walk more during recess	After the walking trail was drawn on the school’s playground, the physical education teacher explained the students about the importance of being physically active and how the students could use the walking trail to be more active during recess.

^a^The “ACTIVITAL” trial aimed at improving diet and physical activity. This table only summarizes the physical activity and screen-time components of the trial by stages, which aimed at improving both physical activity and sedentary behaviour. The pedagogic material of ACTIVITAL trial is available on http://ucuenca.edu.ec/la-investigacion/unidades-de-investigaci%C3%B3n/vlir-uos/programas/nuthealth/servicios

During the second stage, the individual strategy was geared towards ways to overcome the barriers for being physically active. Similar to the first stage, the environmental strategy included a parental workshop with similar topics as the classes with adolescents (e.g. on how to overcome the barriers for being physically active). Also, the environmental strategy included the set-up of a walking trail that was drawn on the floor of the schools. (Table 1). In other words, whereas the first stage of the intervention programme included specific strategies to reduce screen-time behaviour, the second stage did not include these. The table 1 presents more details of the intervention activities.

Both intervention and control schools received the standard physical education curriculum as determined by the Ecuadorian government. This curriculum was geared at increasing sports skills and schedules a mandatory 80 min of physical education/week. It does not deal with sedentary behaviour.

#### Screen-time assessment

Screen-time behaviour was assessed using a validated self-reported questionnaire [22]. Adolescents reported the number of hours that they usually spent watching TV, playing videogames or using the computer on a week- and weekend day. Response categories were “zero”, “30 min”, “1 h”, “2 h”, “3 h”, “4 h”, “5 h” and “>6 h”. In the present study, we report the intervention effect for i) time spent watching TV, playing videogames and using the computer on a week- and weekend day separately, ii) the total amount of screen-time on a week- and on a weekend day which was calculated by summing the time spent watching TV, playing videogames and using computer on a week- and on a weekend day, and iii) the proportion of adolescents that spend more than two hours on an average day on screen-time behaviours (average day = ((total screen-time on a week day*5) + (total screen-time on a weekend day*2))/7). Except for the last outcome, all outcomes were considered as continuous variables. We assessed time spent watching TV, playing videogames or using the computer as these are preferred sedentary behaviours for the Ecuadorian adolescents [19]. Reporting the three screen-time activities separately allows investigating if a possible decrease in one activity is not compensated by an increase in another activity. [6]. In addition, we analysed the intervention effect on weekend days separately because adolescents have a different time structure during a weekday compared to a weekend day [23]. Finally, we reported the total screen-time and the proportion of adolescents who do not reach the international recommendation of screen-time (<2 h/day) [24] in order to facilitate comparisons with similar studies.

#### Other measurements

Baseline BMI z-score and socio-economics status were used as covariates in the statistical analysis. The BMI z-score by age and gender were calculated using the Antro plus software (version 3.2.2, WHO Geneva, Switzerland). The weight and height of adolescents were assessed using a calibrated balance (SECA 803, Hamburg, Germany) and mechanical stadiometer (PORTROD, Health o Meter, Illinois, USA) respectively. Adolescents were measured with light clothing and without shoes in a separate room by a researcher of the same sex.

The socio-economic status (SES) of the adolescent’s household was defined according to the Integrated Social Indicator System for Ecuador [25]. The system classifies a household as “poor” when one or more deprivations related to housing facilities, basic urban services, money, education and physical space are reported otherwise the household is classified as “better-off”. A deprivation of housing facilities is defined when the roofs’ or walls’ material is either cardboard, pieces of aluminium, bamboo, plastic, or any other residual material. A deprivation of urban services appears if the household has precarious or no access to potable water or the house is not connected to a proper sewage system. There is a monetary deprivation if the ratio between household’ members with job over the total members living in the household is higher than 1:3 or when the head of the household (in the economy context) has maximum two years of primary education. There is a deprivation of education if one or more of the children at school age (between 6 and 12 year old) do not attend school. There is deprivation of physical space if the average of persons per bedroom available in the house is higher than three persons/room.

#### Intervention period

The ACTIVITAL project started in October 2009 and ended in June 2012. It took place during the three academic years 2009–2012 with a total duration of 28 months. In the academic year 2009–2010, we conducted the baseline measurements (October 2009 - February 2010) and applied the Intervention Mapping and Comprehensive Participatory Planning and Evaluation protocol (March-June 2010). In the academic year 2010–2011, the first stage of the intervention (September 2010 - February 2011) was implemented and we performed an intermediate follow-up (March-June 2011). In the academic year 2011–2012, the second stage of the intervention was implemented (September 2011- January 2012) and final measurements were performed (from February 2012 - June 2012).

#### Statistical analysis

Data were analysed using Stata (version 12.0, Stata Corporation, Texas, USA). The baseline characteristics were expressed in mean (standard deviation) accounting for cluster design (“svy” Stata command) and in percentages.

Multilevel mixed models with a significance level of 5 % were used to assess the baseline differences (crude models) and intervention effects (full models) on screen-time. Intention-to-treat analysis was performed to assess the intervention effect on the overall period of the trial and after both first and second intervention stage. The fixed part of the full mixed models included the baseline covariates BMI z-score, socio economic status and gender, while the pair matching and subject were assigned as a random part of the model. The Akaike-Schwartz criteria were used to determine the optimal covariance structure and a likelihood ratio test was used to compare the fit of the models. To assess the effect of the adjusting we also analysed the intervention effect by using crude model. The reported intervention effect corresponds to the difference in means for continuous dependent variables. For the dichotomous variables the reported intervention effect represents the difference in absolute risks i.e. the difference in the proportions [26] after the different stages of the intervention.

The mixed model used to analyse the intervention effect after 28 months included the variable “time” and the interaction term “time X group of allocation” as covariates. The variable “time”, measured in months, was the specific time when each observation was collected i.e. it varies from 0 to 31,6 months. In addition, “time” was added as a random slope at level of the measurement. The beta coefficient of the interaction term “time X group of allocation” represents the intervention effect on the outcome over a unit of time (month) [27]. To obtain the intervention effect, the beta coefficient was multiplied by 28 as this was the mean time of measure at second follow-up. The overall intervention effect was analysed considering the three points of measurements. We equally analysed the intervention effect considering only the baseline and last follow-up measurements to obtain a complete understanding of the variation of the data after 28 months.

A mixed model with spline regression was used to analyse the intervention effect by stage, this technique allows to fit multiple linear models to the data for different ranges of time [27]. The knot (the point of time where the slope of the linear function change) was set at 18 months since it was the mean time of measurements at first follow-up. The Stata command “mkspline” allows performing the spline regression by means of the creation of two auxiliary variables, “time1” and “time2”. These auxiliary variables were generated based on the knot and the values of variable “time”. The auxiliary variables time1 is equal to variable “time” when “time” < 18 and equal to 18 when “time” > =18, while “time 2” is equal to zero when “time” is <18 and equal to“time”-18 when “time” > =18. The variables time1, time2 were added as covariates and as a random slopes. The interaction terms “time1 X group of allocation” and “time2 X group of allocation” were added as covariates. The beta coefficient of interaction terms “time1 X group of allocation” and “time2 X group of allocation” represent respectively the intervention effect over a unit the time (months) during the first and second stage of intervention. To obtain the intervention effect after stage 1 and stage 2, the beta coefficients were multiplied by 18 or 10 respectively as these were the mean time of measures at first and second follow-up.

## Results

### Descriptive statistics

In total, 1370 adolescents (control group *n* = 684) from 20 schools (control group *n* = 10) completed the screen-time questionnaires at baseline (mean age 12.8 ± 0.8 years old, 62.4 % girls, mean BMI z-score 0.31 ± 1.05). The time spent watching TV, playing videogames or using the computer and the total screen-time on a weekday and weekend day was not significantly different between the intervention and control group at baseline. At 18 and 28 months of follow-up, 1228 (mean age 14.3 ± 0.8 years old, 62.4 % girls) and 1078 (mean age 15.2 ± 0.8 years old, 63.2 % girls) adolescents completed the questionnaire respectively. No school left the trial. The percentage of adolescents who spend more than 2 h/day on screen-time behaviour increased from 66 % at baseline to 92 % after 28 months in control groups, while in the intervention groups it increased from 69 to 90 % (Table 2)**.** Mean screen-time at baseline was lower compared to that of the two follow-ups (all P-values <0.001).

**Table 2:** Average time spent on watching television, playing videogames and computer use by week and weekend^a^

	Baseline		After 18 months		After 28 months	
	Intervention	Control	Intervention	Control	Intervention	Control
	(*n* = 686)	(*n* = 684)	(*n* = 616)	(*n* = 608)	(*n* = 547)	(*n* = 531)
Screen-time	mean (SD)	mean (SD)	mean (SD)	mean (SD)	mean (SD)	mean (SD)
TV on a weekday (min/day)	100.10 (81.13)	98.20 (86.01)	117.61 (90.11)	128.39 (96.73)	126.36 (96.67)	122.09 (99.14)
TV on a weekend day (min/day)	162.90 (109.23)	162.06 (112.33)	179.12 (113.93)	196.57 (116.22)	181.26 (119.27)	190.00 (114.02)
Video games on a weekday (min/day)	14.13 (34.17)	15.39 (42.69)	18.46 (46.41)	20.08 (47.82)	26.93 (67.99)	20.90 (46.04)
Video games on a weekend day (min/day)	34.50 (62.74)	36.18 (67.33)	45.63 (81.02)	50.83 (83.52)	47.71 (83.80)	50.56 (81.68)
Computer on a weekday (min/day)	50.86 (53.83)	51.67 (62.95)	101.41 (86.77)	98.58 (87.58)	133.00 (107.25)	122.26 (100.95)
Computer on a weekend day (min/day)	52.17 (71.7)	55.26 (75.74)	112.94 (103.46)	118.38 (110.07)	142.82 (117.92)	142.49 (114.63)
Total screen-time on a weekday (min/day)	165.09 (115.07)	165.26 (132.45)	237.32 (151.62)	246.36 (153.15)	286.29 (184.67)	265.25 (164.85)
Total screen-time on a weekend day (min/day)	249.58 (171.27)	253.51 (181.98)	337.69 (206.27)	365.78 (209.09)	371.79 (214.91)	383.05 (211.68)
Percentage of adolescents who exceed 180 min/day on screen-time behaviours	68.9 %	66.5 %	86.3 %	89.2 %	89.6 %	91.7 %

*TV* Television

^a^Standard deviation adjusted for clustering

### Overall intervention effect

Throughout the three measurements, the change in TV time (β = −14.8 min, *P* = 0.02), total screen time on a weekend day (β = −25 min, *P* = 0.03) and the proportion of adolescents that did not reach the recommended screen-time (β = −6 percentage point, *P* = 0.01) were significantly different between adolescents from the intervention and control group (Table 3). To further investigate these changes and directions, we report the intervention effect after stage 1 and 2 (see below).

**Table 3:** Intervention effect on screen-time behaviours per intervention stage and the whole intervention period

Screen-time	Intervention effect after first intervention stage (0 to 18 months)^a^		Intervention effect after second intervention stage (18 to 28 months)^a^			Intervention effect considering baseline and last follow-up measurements^d^			Overall intervention effect considering three measurements^e^		
	β [95 % CI]	P^b^	β [95 % CI]	P^b^	ICC^c^	β [95 % CI]	P^b^	ICC^c^	β [95 % CI]	P^b^	ICC^c^
TV on a weekday (min/day)	−15.66 [−26.1, −5.22]	0.003	13.10 [2.00, 24.20]	0.02	0.08	−2.52 [−13.44, 8.68]	0.66	0.07	−7.84 [−17.92, 2.52]	0.14	0.08
TV on a weekend (min/day)	−18.90 [−32.04, −5.58]	0.005	9.80 [−4.50, 24.10]	0.18	0.00	−10.36 [−23.8, 2.8]	0.12	0.00	−14.84 [−27.44, −2.52]	0.02	0.00
Video games on a weekday (min/day)	−0.72 [−5.94, 4.32]	0.76	5.90 [−1.00, 12.80]	0.10	0.00	5.88 [−0.84, 12.6]	0.08	0.00	3.92 [−1.68, 9.52]	0.18	0.00
Video games on a weekend day (min/day)	−1.80 [−10.44, 6.84]	0.68	0.30 [−9.20, 9.70]	0.96	0.01	−3.08 [−11.76, 5.6]	0.50	0.01	−2.80 [−10.64, 5.32]	0.52	0.01
Computer on a weekday (min/day)	1.80 [−8.64, 12.24]	0.74	3.10 [−8.10, 14.20]	0.59	0.08	3.92 [−8.4, 16.52]	0.52	0.06	4.48 [−7.84, 16.8]	0.48	0.07
Computer on a weekend day (min/day)	−3.42 [−16.38, 9.54]	0.60	0.30 [−12.70, 13.30]	0.96	0.08	−6.44 [−20.72, 7.84]	0.37	0.07	−5.04 [−19.32, 8.96]	0.48	0.07
Total screen-time on a weekday (min/day)	−14.22 [−30.78, 2.52]	0.10	21.40 [2.10, 40.70]	0.03	0.04	7.22 [−12.88, 27.44]	0.48	0.03	0.56 [−17.64, 19.04]	0.95	0.03
Total screen-time on a weekend day (min/day)	−25.92 [−48.96, −3.06]	0.03	9.60 [−14.40, 33.60]	0.43	0.02	−22.42 [−47.04, 2.24]	0.07	0.02	−25.20 [−47.88, −2.8]	0.03	0.02
Proportion of adolescents who exceed 180 min/day on screen-time behaviours	−0.04 [−0.09, 0.01]	0.01	0.00 [−0.05, 0.05]	0.98	0.00	−0.05 [−0.10, 0.00]	0.06	0.01	−0.06 [−0.10, −0.01]	0.01	0.00

*TV* Television

^a^The spline regression was used to assess the effect of intervention effect over the time. The knot was the mean time for the first follow-up (18 months)

^b^The models were adjusted by adolescents’ BMI z-score, socio-economic status and gender

^c^Intracluster correlation coefficient

^d^The multilevel modeling included the measurements at baseline and follow-up at 28 months

^e^The multilevel modeling included the measurements at baseline, follow-up at 18 months and follow-up at 28 months

### Intervention effect between 0 and 18 months (first stage of the intervention)

TV time on a weekday (β = −15.7 min; *P* = 0.003) or weekend day (β = −18.9 min; *P* = 0.005), total screen-time on a weekend day (β = −25.9 min; *P* = 0.03) and the proportion of adolescents that did not meet the screen-time recommendation (β = −4 percentage point; *P* = 0.01) increased less in the intervention group compared to the control group after 18 months (Table 3).

### Intervention effect between 18 and 28 months (second stage of the intervention)

There was a significant intervention effect for TV on a weekday (β = 13.1 min; P = 0.02). For this outcome the intervention effect was in favour to the control schools. TV time on a weekday increased in the intervention group while it decreased in the control schools. In addition, total screen-time on a weekday (β = 21.4 min; *P* = 0.03) increased more in the intervention schools compared to the control schools (Table 3).

### Intervention effect between 0 and 28 months

There was no difference in the change in screen-time behaviour between baseline and second follow-up between adolescents from intervention and control schools (Table 3).

### Sensitivity analysis

Results from unadjusted and adjusted models differed only for one outcome, the proportion of adolescents that did not meet the screen-time recommendation. For this outcome the intervention effect on the first stage changed from significant (β = −4 percentage point; *P* = 0.01) to non-significant (β = −5 percentage point; *P* = 0.06) for unadjusted model. While, when baseline and second follow-up were only considered the proportion of adolescents that did not meet the screen-time recommendation changed from non-significant (β = −5 percentage point; *P* = 0.06) to significant (β = −6 percentage point; *P* = 0.02) for unadjusted model (Table 4).

**Table 4:** Unadjusted models for intervention effect on screen-time per intervention stage and the whole intervention period

Screen-time	Intervention effect after first intervention stage (0 to 18 months)^a^		Intervention effect after second intervention stage (18 to 28 months)^a^			Intervention effect considering baseline and last follow-up measurements^d^			Overall intervention effect considering three measurements^e^		
	β [95 % CI]	P^b^	β [95 % CI]	P^b^	ICC ^c^	β [95 % CI]	P^b^	ICC ^c^	β [95 % CI]	P^b^	ICC ^c^
TV on a weekday (min/day)	−14.81 [−25.09, −4.52]	0.005	11.96 [1.08, 22.84]	0.03	0.08	−3.00 [−13.72, 16.35]	0.58	0.07	−7.84 [−17.95, 2.27]	0.13	0.08
TV on a weekend (min/day)	−18.32 [−31.45, −5.18]	0.006	9.93 [−6.2, 26.06]	0.23	0.00	−10.22 [−23.24, 3.44]	0.12	0.00	−15.15 [−27.33, −2.97]	0.02	0.00
Video games on a weekday (min/day)	−1.89 [−7.07, 3.28]	0.47	5.96 [−0.77, 12.68]	0.08	0.03	5.068 [−1.68, 3.95]	0.14	0.03	2.80 [−3.22, 8.79]	0.36	0.03
Video games on a weekend day (min/day)	−6.08 [−14.89, 2.72]	0.18	1.51 [−7.79, 10.81]	0.75	0.04	−5.18 [−14.28, 7.42]	0.27	0.04	−6.50 [−15.01, 2.04]	0.13	0.05
Computer on a weekday (min/day)	2.52 [−7.85, 12.89]	0.63	2.16 [−8.89, 13.21]	0.70	0.09	3.00 [−9.24, 17.67]	0.63	0.07	4.48 [−7.78, 16.74]	0.48	0.08
Computer on a weekend day (min/day)	−4.19 [−17.03, 8.64]	0.52	0.73 [−12.14, 13.6]	0.91	0.09	−6.50 [−20.72, 10.30]	0.37	0.08	−5.38 [−19.43, 8.65]	0.45	0.08
Total screen-time on a weekday (min/day)	−13.45 [−29.81, 2.92]	0.11	19.3 [0.4, 38.21]	0.05	0.04	5.15 [−14.56, 17.02]	0.61	0.03	−0.64 [−18.68, 17.39]	0.94	0.03
Total screen-time on a weekend day (min/day)	−30.42 [−53.17, −7.69]	0.01	11.97 [−11.54, 35.47]	0.32	0.02	−23.24 [−47.60, 1.74]	0.06	0.02	−28.42 [−50.74, −6.10]	0.01	0.02
Proportion of adolescents who exceed 180 min/day on screen-time behaviours	−0.05 [−0.18, 1.10]	0.06	0.00 [−0.1, 0.0]	0.90	0.01	−0.06 [0.0, 0.0]	0.02	0.02	−0.06 [0.0, 0.0]	0.004	0.01

*TV* Television

^a^The spline regression was used to assess the effect of intervention effect over the time. The knot was the mean time for the first follow-up (18 months)

^b^The models were not adjusted

^c^Intracluster correlation coefficient

^d^The multilevel modeling included the measurements at baseline and follow-up at 28 months

^e^The multilevel modeling included the measurements at baseline, follow-up at 18 months and follow-up at 28 months

## Discussion

The present study reported the effect of a comprehensive school-based health promotion intervention on screen-time on Ecuadorian adolescents. The results showed that the change over the three measurement periods in TV time, total screen-time and the proportion of adolescents who did not reach the screen-time recommendation differed between adolescents from the intervention and control schools. To fully understand the direction of this change and since the two intervention stages had a different focus, we specifically looked at the effect after the first and the second stage. In general the intervention effect after the first stage was in favour of the adolescents in the intervention group, whilst in the second stage the intervention effect was in favour of the adolescents in the control group. If we compared the screen-time behaviour at baseline and the second follow-up measurement, there was no difference in the change in screen-time between the two groups.

After the first stage of the intervention, adolescents from the intervention schools had a smaller increase in TV time and total screen-time behaviours compared to the adolescents from the control schools. The proportion of adolescents that reached the screen-time recommendation decreased significantly less in the intervention group. Previous studies have shown that as adolescents get older, they spend more time in front of a screen [28]. Our findings suggest that the intervention programme was able to mitigate the increase in adolescents’ TV and screen-time. These positive results could be a result of the programme’s emphasis on decreasing screen-time behaviour with a particular focus on TV time during the first stage of the intervention programme. Our positive results are in line with other school-based intervention programmes focusing on different health behaviours including screen-time behaviour [13, 14, 16], namely The Planet Health programme [16] and the DOiT [13] programme. Both interventions aimed to improve dietary, physical activity and screen-time behaviours, and implemented an individual classroom-based intervention. The Planet Health did not implement the environmental component in contrast to the DOiT programme and our intervention. Our findings hence confirm that focusing on screen-time behaviour in combination with other health behaviours might result in an effect on screen-time [10].

During the second stage, we did not observe the positive effect on adolescents’ screen-time anymore. Indeed, in contrast to the first stage, adolescents from the intervention schools had a larger increase in TV time and total screen-time than adolescents from the control schools in the course of the second stage. This could be attributed to the fact that during the second stage of the programme, the intervention contained no specific strategies on the reduction of screen-time behaviours [10], and focused essentially on improving diet and physical activity. This finding is in line with the conclusion that reducing screen-time is only possible when the intervention focuses specifically on reducing screen-time.

The initial slower increase of screen-time for the adolescents from the intervention school after stage 1 was followed by a stronger increase of screen-time. As a result there was no difference in the intervention effect on screen-time when only the baseline and second follow-up measurement were considered. This means that there was an effect on screen-time when the intervention included activities focused on screen-time, but when the intervention is focused on other health behaviours, adolescents returned to their old behaviour. This could be due to the fact that screen-time behaviour has a strong habitual nature that is difficult to change [6]. Therefore, a continuous focus on the reduction of screen-time behaviour might be required. In Ecuadorian adolescents specifically, the topic of reducing screen-time behaviour for health is new and requires continuous attention.

The intervention effects, in favour to the intervention group, after the first stage of the intervention on TV time are relevant as watching TV is the preferred leisure-activity for Ecuadorian adolescents [19]. In addition, TV time represents one-third of the sedentary time of a typical adolescent, and those who watch more TV are more likely to have unhealthy dietary patterns [29] and lower self-esteem and to be more aggressive [1]. We did not find differences on the time spent on playing video games or using computer between the adolescents from the intervention and control groups over the whole period. We attribute this result to the fact that the intervention programme did not include specific strategies to decrease the time spent on playing video games or using computer. Also, the findings suggest that there was no compensation phenomenon i.e. the time decreased for TV time was not allocated to other screen-time behaviours.

Finally, we found that in the present study 67 % of all adolescents spend more than 2 h/day on screen-time at baseline, and that this percentage increased during the adolescents’ transition from 12 to 14 years (up to 87 %). After 28 months, the percentage increased up to 92 %, therefore it is important to put continuous effort into developing intervention programmes aiming to reduce screen-time in adolescents in Latin-America.

The present study has several strengths. First, to our knowledge this is the first study that reports the effect of a school-based health promotion intervention programme on screen-time behaviours among adolescents from a low- or middle-income country. Second, the sample size is relatively large, considering that most (70 %) of the studies targeting sedentary behaviours in schools have less than 500 participants [6]. Third, the duration of our intervention was longer than other comparable intervention studies [6]. A final strength is that three time points were included to investigate the effect of the ACTIVITAL intervention. By doing so, we were able to analyse the intervention effect on screen-time after stage 1 of the intervention. We note that no intervention effect would have been found if only the first and last time point would have been included in the analyses. This finding illustrates the need for multiple measurements to document the effect of similar interventions.

A limitation of this study is that the screen-time was self-reported and could be subject to social desirability. Also, although our results are encouraging for school interventions in LMICs, our findings are both mixed and modest, since the intervention effect was limited to minimize the increasing of screen time on the first stage. Therefore, there is still a needed for studies focused on decrease the screen-time among adolescents from LMICs Additionally, the results of the present manuscript are limited to the populations with similar characteristics to Ecuadorian adolescents i.e. mixed *mestizo* ethnicity and living in urban areas at high altitude.

## Conclusion

The ACTIVITAL programme, a school-based intervention aimed at promoting healthy diet, physical activity and screen-time behaviour, was able to mitigate the increase in adolescents’ TV time and total screen-time. The effect was observed mainly after the first stage of the intervention, which focused on decreasing screen-time behaviour. However, after the intervention strategies for reducing screen-time behaviour finished, the adolescents from the intervention group increased the screen-time again.

## Abbreviations

Min: Minutes
TV: Television
